# A Comparative Study of Growth Patterns in Crested Langurs and Vervet Monkeys

**DOI:** 10.1155/2011/948671

**Published:** 2011-02-21

**Authors:** Debra R. Bolter

**Affiliations:** Department of Anthropology, Modesto College, 435 College Avenue, Modesto, CA 95350, USA

## Abstract

The physical growth patterns of crested langurs and vervet monkeys are investigated for several unilinear dimensions. Long bone lengths, trunk height, foot length, epiphyseal fusion of the long bones and the pelvis, and cranial capacity are compared through six dental growth stages in male *Trachypithecus cristatus* (crested langurs) and *Cercopithecus aethiops* (vervet monkeys). Results show that the body elements of crested langurs mature differently than those of vervets. In some dimensions, langurs and vervets grow comparably, in others vervets attain adult values in advance of crested langurs, and in one feature the langurs are accelerated. Several factors may explain this difference, including phylogeny, diet, ecology, and locomotion. This study proposes that locomotor requirements affect differences in somatic growth between the species.

## 1. Introduction

The fundamentals of growth and development are important in primate evolutionary studies. Primates have a protracted period of immaturity compared to other animals, and immature individuals must survive this prolonged life period before reproductive adulthood. When considering a life history perspective, natural selection acts on immature individuals first through survival, when mortality is quite high; only when an individual successfully navigates the long infant and juvenile stages does reproduction become a selective force. Emphasis on primate survival through immaturity and life history theory began in the 1980s and has become a major focus for primate researchers: we assess basic demographic variables like group composition and age at reproductive maturity, establish age-cohorts, follow changes in individuals as they mature, investigate patterns of sexual dimorphism, examine the influence of ecological and locomotor constraints, and explore maturity disassociations among body systems [1–16]. In order to determine the life history parameters for extinct species, paleontologists and paleoanthropologists must understand growth and development (e.g., [17–21]). Evolutionary theorists use displaced developmental events (from ancestor to descendant) to elucidate patterns of adaptations (heterochrony) or shifts in multidimensional, shape features (allometry) (e.g., [22, 23]). A new term, sequence heterochrony, has been applied to investigations of the shift in developmental sequences from ancestor to descendants—a way to confer change (evolution) without novel traits (e.g., [24, 25]). 

Long-term field studies on known individuals facilitate the correlation of an equivalent growth event: for example, juvenile independence with dental development and social maturation with reproductive anatomy (e.g., [2, 26]). Much of the long-term fieldwork in Old World monkeys comes from the cheek-pouched cercopithecines (e.g., [11, 27–30]); somewhat less information is available for the colobines (e.g., [31]). Skeletal materials in museums provide additional information on the life history events in Old World monkeys, for example, in the correlation between the emergence of M1 and 90%–95% of brain growth [32].

Although wild, naturally ranging primates are the preferred focus for developmental studies, nevertheless, life history stages for captive animals may be conducted using techniques not easily derived from wild populations. Colony animals permit longitudinal monitoring, which provides species data on dental development, skeletal maturity, body masses, and reproductive physiology (e.g., [33–40]). However, as the circumstances of growth differ between wild and captive individuals, the timing of development differs in the two settings (e.g., [10, 41–45]).

This study provides a first step to consider body maturity in wild-collected, local populations of two Cercopithecidae: Asian crested langurs (*Trachypithecus cristatus*) and African vervet monkeys (*Cercopithecus aethiops*).  Table 1 compares the life history variables and their species traits. In both species, the length of gestation is the same, between 5.5 to 6 months; the first birth occurs around 4 years whereas males become reproductively mature at about 5 years. Physical markers of life history are comparable, with first molar (M1) emergence at 1 year of age and third molar (M3) emergence as early as 3 years in both species, with no notable sex differences in timing. Male crested langurs average 6.6 kg, and male vervets 5.8 kg. 

This study investigates and compares the development of long bone length, brain size, skeletal fusion, and body mass between langurs and vervets. To control for the time dimension, molar eruptions are used to standardize the comparisons. Males are the focus because female vervets are lighter than female langurs. 

I test the hypothesis that body systems (brain, skeleton, and linear dimensions) in male crested langurs grow the same as that of male vervets. By holding dental growth constant, the evaluation of the other systems is possible. Dental development thus becomes the chronological marker for comparison and contrast of crested langurs and vervets at critical stages of life—almost anatomical “snap-shots.”

## 2. Methods

Thirty-three male *Trachypithecus cristatus *(17 immature, 16 adult) were wild shot in the 1937 Asiatic Primate Expedition (A.P.E.) in North Boreno under the direction of Adolph Schultz and assisted by Sherwood Washburn (cf [62]). Ten years later Washburn collected 35 male *Cercopithecus aethiops* (13 immature, 22 adult) in Uganda over a 35-day period. Individuals for each population span all immature age classes, although the sample sizes are small.

For both species, each animal was weighed at once, linear measurements were taken, and external features noted (see Table 2). Field notes recorded the sex (visual assessment), measurements on trunk height (symphysion to suprasternal notch), length of tail and foot, and body mass (anthropometrics after [63]). 

In the laboratory dental eruption, epiphyseal and pelvic fusion were assessed. Maximum lengths of the limb bones were measured after Schultz [63]. Cranial capacity, an indirect indicator of brain size, was measured with mustard seed and the volume recorded to the nearest milliliter (see Table 2).

### 2.1. Animal Sample: Dental Age Classes for Basis of Life Stages

Age classes in the two species are defined by molar emergence. Those individuals without permanent dentition were classified as “infant,” those with fully erupted molars and fused proximal humeri as “adult”, and the intermediaries as “juvenile” (see Table 3). 

Analysis of dentition is the standard method by which to organize age classes across species (e.g., [67]). In colobines, the posterior molar teeth or “new” teeth erupt before their anterior incisor and canine ones, or “replacement” teeth (e.g., [46, 68, 69]). This pattern contrasts with noncolobines, in which the posterior teeth emerge later in the sequence [68, 70]. Individuals in this study are classified based on permanent molars emergence. For all primates M1 erupts after deciduous dentition has erupted, followed by the M2 and M3. These categories are limited to molar eruptions and therefore allow refinement of juvenile life stages, thus eliminating the problem introduced by variations in anterior/posterior eruption schedules across larger taxonomic divides.

Dental emergence chronologies from live animals have been documented for both species to serve as supplemental data for this analysis. Gingival emergence is slightly later than the emergence through the alveolar bone and therefore the chronologies in Table 3 may overestimate the age of the skeletal age classes up to 3 months [71]. 

Wolf [49] documents gingival emergence times of wild crested langurs that correspond with the predicted timing sequences published by Smith et al. [70], Godfrey et al. [12], and Dirks [13] for African and Asian colobine species. These times are found in Table 3. For the vervets, gingival emergence times come from two vervet colonies on individuals of known age [33, 40]. The vervet age classes are revised from a previously published paper to allow comparison with the langurs [60]. Note that since dental growth is faster in captive versus wild primates, wild vervets' ages may be underestimated by as much as 9 months (based on M3 eruption in captive baboons 18 months earlier than in their wild counterparts [41], and baboons requiring twice the time to mature as vervets).

Eruptions of maxillary teeth for this study were visually assessed and assigned scores based on Wintheiser et al. [72] (1 = unerupted, 2 = partial  eruption, 3 = full  eruption). The upper dentition was chosen because these teeth were most often preserved as part of the cranium, and therefore maximized the specimens for study. 

Skeletal fusion stages of the long bones and pelvis were assigned according to Wintheiser et al. [72] methods (1 = no union-two pieces of bone with no connection, 2 = partial union-bony connection between bone elements, with an opening between them, 3 = full union-complete fusion, no opening). Study skeletons were fully macerated so that the bones were free of cartilaginous tissue, except in the cases of newborns. The ossification event, once initiated, occurs quickly [73] which makes classification into the three categories unambiguous. Postcranial markers are added to the age class categories in order to separate immature from adult since the dentition completes growth before the postcrania (e.g., [46, 74]). The proximal humerus is the last long bone to fuse in monkeys [75], and this skeletal element divided immature from adult and serves as a marker for use across taxa (e.g., [44, 60]).

### 2.2. Data Analysis to Compare Sequence and Timing of Growth

Each individual was (1) scored for dental emergence and skeletal fusion, (2) assigned a dental age class, and (3) measured for cranial capacity, skeletal fusion, and limb bone lengths. In evaluating the fusions of skeletal elements, the most advanced fusion state was taken per age class.

#### 2.2.1. Analyzing Order of Body Growth

To configure the percentage dental emergence completed in juveniles, eruption scores were added together and divided by the scores for adults (after [60]). To configure overall skeletal maturity in juveniles, the following 19 epiphyseal union scores were added together and divided by the scores for adults: proximal (*p*), medial, and distal (*d*) humerus; *p* and *d* radius, ulna, tibia, fibula, femur; greater and less trochanter of femur; three borders of the acetabulum and the ischiopubic ramus.

### 2.3. Statistical Analysis

Descriptive statistics were taken for juvenile and adult age classes. Analysis of variance (ANOVA) was calculated for linear and mass measurements across age classes 3, 4, and 5 to be compared with the adult stage, age class 6. *P* ≤ .05 was taken as significant.

## 3. Results

### 3.1. Skeletal Growth Patterns: Epiphyseal Fusions

Fusion that begins at the ischiopubic ramus in the infant is completed by class 2 (see Table 4). Fusion within the acetabulum begins in vervets during class 3, in langurs class 4 and is completed during class 5 in both species. 

In both species, fusion of the long bones begins at the elbow joint in class 3 but the sequence of fusion varies between species. In langurs, most skeletal elements fuse after M3 emergence, class 5, whereas vervet males have a more mosaic skeletal maturity from class 3–5 (see Table 5).

### 3.2. Skeletal Growth Patterns: Linear Dimensions

Crested langurs in class 5 compared to adults (age class 6) have statistically significantly shorter linear dimensions for all measurements except for the tail and femur. In terms of ranges, two “small” adults consistently fall within the age class 5 individual ranges (*n* = 3) for the humerus, radius, tibia, and trunk length, while the majority of adults fall outside of the range (*n* = 14). 

**Figure 1:**
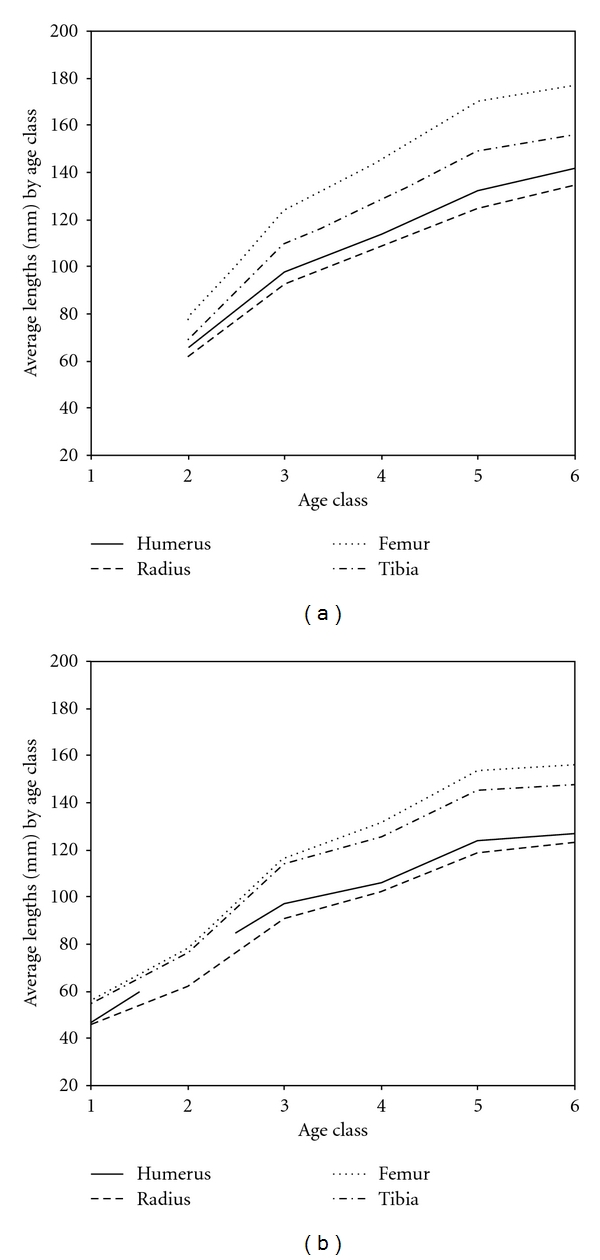
Average maximum long bone length (mm) in relation to age class for (a) langurs and (b) vervets.

**Figure 2:**
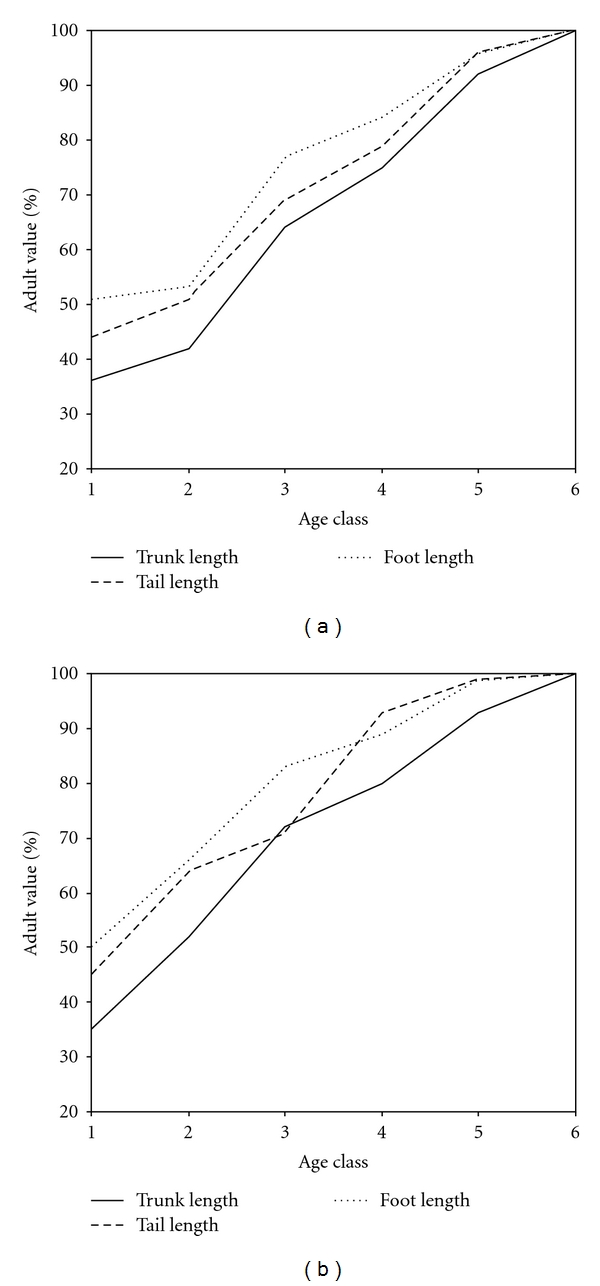
Relative lengths in mm (against adult values) in relation to age class for (a) langurs and (b) vervets.

In vervets, all long bones, the tail and the foot in age class 5 animals are not statistically different from adult lengths, whereas the trunk height in age class 5 is significantly shorter (see Table 6, Figures 1 and 2). When considering the range of adult values, one lone age class 6 male has a trunk length of 310 mm while the age class 5 males measure 320, 320, and 326; all other adult males (*n* = 19) have trunks that range from 330–376 mm.

### 3.3. Body Growth Patterns: Mass Dimensions

**Figure 3:**
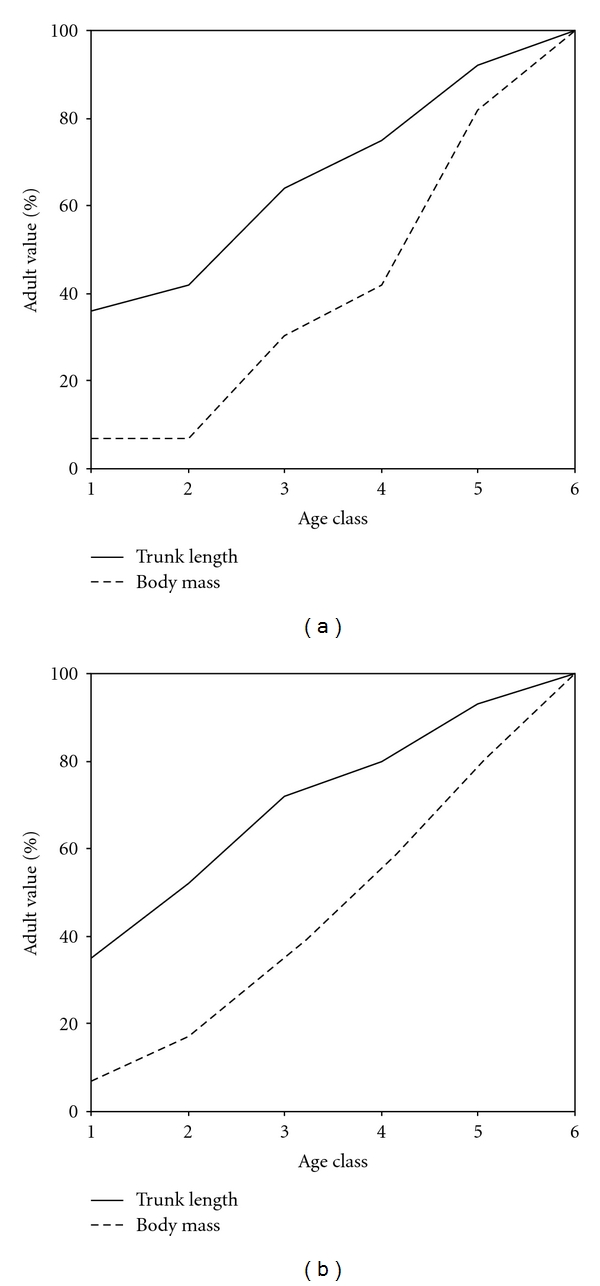
Percent body mass and trunk height attained by age class for (a) langurs and (b) vervets.

In both species, cranial capacity reaches over 90% completion by 1st molar eruption (class 2) and body mass in late juveniles (class 5) falls significantly below the range of adults (see Figure 3). In langurs, age class 5 males range 5.0–5.9 kg, while age class 6 adults range 5.7–7.9 kg. In vervets, age class 5 males range 3.9–5.4 kg, while adults range between 4.1–7.3 kg.

### 3.4. Order of Growth in Body Systems

Crested langurs and vervets show the same *order* of the maturation in body dimensions, proportions, dentition, and skeleton (see Table 7). 

### 3.5. Timing of Growth in Body Systems

Table 8 shows the percent adult value for linear and mass dimensions by species. Langur and vervet newborns are proportional in size compared to respective adult values. In age class 2–5, langurs are smaller (compared to adults) than vervets. The one exception is age class 5 body mass.

## 4. Discussion

This study offers a focused comparison between two Old World monkeys to establish growth patterns in several unilinear body dimensions. Further detailed studies on primate growth are required to ascertain which sequence reflects the ancestral condition of the Cercopithecoidea, and therefore this study precludes a heterochronic analysis (cf. [23]). Likewise, as multidimensional shape changes are not being investigated, allometric scaling cannot be applied to these data. 

### 4.1. Similarities between Crested Langurs and Vervets

The *order* of maturity of body systems between crested langurs and vervets is consistent (refer to Table 7). The brain matures first, then the linear dimensions of the tail, limbs, and finally the trunk. Body mass and epiphyseal closure are the last features to change. This sequence of growth supports the hypothesis of Bolter and Zihlman [60] that the order represents a conservative pattern in catarrhines. Their order of skeletal fusion in the long bones is also similar. The distal humerus is the first to fuse, the proximal humerus one of the last. The forelimb fusion is also consistent between vervets and langurs: elbow elements unite in class 3, with wrist and shoulder elements fusing during class 5. A significant proportion of growth occurs during the late juvenile stage in both species—that is, after all permanent teeth have emerged. For example, males reach adult lengths in the tail and femur during class 5 (langurs: 3–5 years; vervets: 3.2–4.5 years). Adult trunk height is achieved in langurs and vervets in class 6.

### 4.2. Differences between Crested Langur and Vervet Maturity

Vervets initiate fusion in several bone elements before langurs: at the ankle and hip joint and reach adult lengths before langurs in the tail, long bones, and foot. 

Fusion of the elements of the hindlimb and hip is accelerated in vervets relative to langurs. Fusion of the ankle joint occurs over class 2-3 in the vervets, in class 5 in langurs. Hip joint fusion at the acetabulum and proximal femur begins in the vervets about 1 year before langurs. In one element of the hindlimb, the knee joint, the distal femur and proximal tibia of the langurs are accelerated compared with vervets.

The tail matures faster in vervets than in langurs. The tail of the langur 2-3 years old is only 79% of adult values (562.5 mm versus 707.9 mm). The 2.2-3.2-year-old vervets are over 90% of adult size (576.7 mm versus 617.0 mm). However, adult lengths are not reached until class 5 in both species.

Long bone and foot lengths reach adult proportions in class 5 in vervets, whereas in langurs of similar age the humerii, radii, tibiae, and hindfeet are statistically shorter. In the crested langur, some long bones (humerus, radius, tibia) fuse *before* adult lengths are achieved. One explanation is that the bones are remodeled as muscularity increases. In a study that included diaphyseal and maximum length measurements in crested langurs, diaphyseal lengths in class 5 are in the adult range whereas the maximum lengths are not [61]. However, this hypothesis is difficult to test without longitudinal data on live animals, which currently do not exist. Another more probable explanation is that the differences are a byproduct of sampling bias.

Several possibilities may explain the variability in langur and vervet growth, and here I focus on two. (1) Molars emerge faster in colobines (langurs), which makes postcranial growth appear immature compared to vervets. In this scenario, only molar teeth grow more rapidly in langurs, but other body systems would not. However, this dental fast-growth explanation is not supported. Some langur somatic elements mature at the same pace as vervets (forelimbs), some later (hip, ankle, tail), and a few earlier (knee elements).

(2) When postcranial growth is compared by molar eruption (regardless of chronological age in years), the mosaic maturity between two species reflects their separate evolutionary histories and/or adaptations. Several explanations may account for these differences: phylogenetic distance, social organization, diet, feeding ecology, geographic location, and locomotion. Here I propose the hypothesis that locomotor differences in crested langurs and vervets may account for the growth differences.

### 4.3. Differential Growth Rates: A Function of Locomotion

Mammals range from very immature and helpless at birth (altricial) to self-sufficient as neonates (precocial). Using brain and muscular development, Grand [76] separates this developmental continuum into four categories. Altricial neonates have small brains and weak muscles, like the giant panda, 98% of whose growth is postnatal, and who remains nest-bound for the first 3 months of life. In contrast, precocial newborns have large brains and strong muscles, like the wildebeest which moves as an adult (30 mph) a few hours after birth. Primates are intermediate: large brains (about 50% of adult size), receptive and responsive to social complexity in early life, but abjectly helpless with weak muscles [76]. They must cling onto the body hair of an adult for transport, a positional behavior reflected in the heavy hands and feet of infants, and well-muscled forearms [77].

Wild primates, for example, baboons, vervets, macaques, and chimps, compared to their captive counterparts have extended growth periods (baboons: e.g., [2, 41], vervets: [55, 78], macaques: [7], chimpanzees: [26, 42–44, 79, 80]). One explanation for this difference between wild and captive growth is that as individuals grow in wild populations, they have greater energy output in their daily lives [9, 44]. As Altmann so clearly stated based on long-term field studies of baboons, “locomotion probably is the largest energy-consuming activity … for most mammals” [11, page 349]. A recent study on three-toed sloths (*Bradypus variegatus*) documents that wild animals expend more energy foraging and avoiding predators and sleep less than their captive counterparts [81]. 

As juveniles make the transition to moving and foraging on their own, their locomotor repertoire expands. As muscular and other body tissues develop and as energy demands increase, one would expect that as feeding ecology and positional behavior vary between species, so would their somatic development. Specifically in vervets the “faster-maturing” postcranial elements (the hip and ankle joint, hindfoot and tail) accelerate their locomotor independence compared to crested langurs. The hindlimb and tail are critical to terrestrial locomotion and may signal the ability to transition between arboreal and terrestrial movement. The hindlimb is dominant in terrestrial walking and bears considerable weight [82]. During locomotion, the long tail of the vervet is critical in propulsive running, leaping, climbing, and balance [83].

Vervets spend about equal amounts of time on the ground and in the trees to forage. This is the justification for their behavior to be described as “semiterrestrial” [84]. In searching for widely distributed resources like fruit, seeds, bark, insects, leaves, grass, and flowers, they must move 600–800 meters, which constitutes about 30% of their day [84].

In contrast, wild crested langurs spend less than 15% of their day moving, almost exclusively in the trees [49, 85]. These arboreal quadrupeds exhibit less travel then vervets, at 200–500 meters per day [85]. In one species of colobines, the Phayre's leaf monkeys (*T. phayrei*), the juveniles rely mostly on the accessible leaves, in contrast to preferred, unripe fruits of the adults [66]. This reliance on abundant but less preferred, substantive leaves translates into increased foraging time for juveniles, but not necessarily longer travel times. Langur juveniles therefore may not experience the same locomotor selective pressures on their postcranial development, as do the vervets.

### 4.4. Link between Locomotion and Skeletal Maturity

Is there a link between locomotion and postcranial maturity? Dynamic compressive forces accelerate long bone epiphyseal fusion in rats [86]. Support for a functional relationship with the regions of maturation in primates is seen with skeletal elements of each joint region fusing around the same time [73, 87]. Typically the sequence of joint fusion (e.g., hip before knee) is conservative among primates, even given locomotor differences [75, 88, 89]. However, there are exceptions. In chimpanzees and orangutans where upper limbs bear weight during suspensory locomotion, the humeral head is accelerated in fusion sequence before the wrist, whereas in most other primates the shoulder fuses after the wrist epiphysis [73, 75]. In bipedal humans, the ankle and metatarsals of the foot are accelerated in fusion sequence compared with quadrupedal monkeys [73]. 

The *timing* of fusion may also reflect locomotor specializations [61, 89]. In large-bodied leapers like proboscis monkeys (*Nasalis larvatus*) and red-tailed monkeys (*Cercopithecus ascanius*), the ilium-to-ischium bones completely fuse before the other two bones of the acetabulum begin to unite; in quadrupedal monkeys, like vervets and mangabeys (*Lophocebus albigena), *fusion is more uniform across the hip (tri-radiate complex) [61].

This relationship between locomotion and postcranial maturity is supported by data from provisioned, free-ranging rhesus macaques (*Macaca mulatta*) on Cayo Santiago [87]. (Note that methods among this study and the Cayo Santiago study vary slightly). The Cayo Santiago rhesus monkeys over 18 months old are primarily terrestrial (64%) and quadrupedal (71%) with terrestrial/arboreal transitions through leaping (~10%) and climbing (~11%) [90]. The juvenile rhesus monkeys' hip and ankle region fuse similarly to the vervet sample in this study, both of which are accelerated by age class compared to the arboreal crested langurs.

One study on New World monkey saddle-back tamarins (*Saguinus fuscicollis*) shows a possible skeletal maturation pattern consistent with vervets, rather than langurs, although the methods differ from this study [91]. In the elbow joint, tamarins are comparable to both langurs and vervets, fusing during class 3. In the acetabulum/hip joint, tamarins fuse these elements during class 3, similar to vervets, whereas in langurs the fusion occurs later during class 4. The knee region appears consistent with vervets in fusing earlier than the langurs, before the time of proximal humerus fusion, or before class 6. Tamarins range daily about 1140–1590 meters which constitutes 20% of their daily activities [92, 93]. Another 33% of the day is spent on foraging for plant matter and insects, in the trees and on the ground [94]. Yearlings carry infant siblings which likely compounds the selective pressures on their locomotor efficiency [95].

Earlier fusion times in vervets, therefore, may be a complex dynamic of selection for immature individuals with more efficient locomotion coupled with the functional demands of mechanical loads that accelerate fusion of particular joints (cf. [96]). Young vervets must move around to get enough food to survive while the crested langur immatures have far less selective pressure for agile locomotion. This study highlights the need for future longitudinal research focusing on the relationship between growth, development, and locomotion in primates.

## 5. Conclusions

This study on dental and skeletal growth of vervet (cercopithecine) and langurs (colobine) monkeys under natural conditions provides a first step in proposing connections between body growth patterns and locomotion. By focusing on postcranial (e.g., long bone, tail, and body mass) as well as cranial (dentition) features, it is possible to connect maturity to behaviors that are essential for survival, especially foraging, traveling, and locomotion and therefore provide a more holistic and integrated view of growth and development. Phylogeny, diet and ecology, as well as locomotion may also influence timing and patterns of growth within a species and more information on growth and development in wild monkeys as well as longitudinal growth data on captive animals will help interpret the contribution of each variable. This approach may also be useful in interpretations of immature fossils, especially in the case of bipedal locomotion and early hominin ancestors.

## Figures and Tables

**Table 1:** Life history features for *Trachypithecus cristatus *and *Cercopithecus aethiops. *

	Gestation (days)	First birth (years)	Interbirth interval (months)	Birth season?	Life span (years)	Female adult body mass (kg)	Male adult body mass (kg)	Male adult brain size (cc)	Diet	Locomotion
*T. cristatus*	~180	~4	16.5–24	No	UNK	5.8	6.6	67	Mature & young leaves, fruits, flowers	Arboreal quadruped
*C. aethiops*	162	3–5	20.4	Yes	31	3.5	5.8	69	Vertebrates, eggs, snails, corms, bulbs, fruits	Semiterrestrial quadruped

Data from [11, 12, 35, 46–61].

**Table 2:** Available data for the two collections.

Field data	Lab data
Sex	Cranial capacity
Trunk height	Dental emergence
Tail length	Humerus length
Foot length	Radius length
Body mass	Femur length
	Tibia length
	Epiphyseal fusion
	Pelvic fusion

**Table 3:** Age classes for crested langurs and vervets.

Langurs		Age classes			Vervets	
Crested langur sample size	Age in years^1^	Age class	Life stage	Tooth emergence (Maxilla)	Age in years^2^	Vervet sample size
2-♂	0–.4	1	Infant	Partial deciduous	0–.3	2-♂
2-♂	.4–1	2		Deciduous	.3–1	2-♂
7-♂	1-2	3	Juvenile	M1	1–2.2	2-♂
3-♂	2-3	4		M2	2.2-3.2	4-♂
3-♂	3–5^3^	5		M3	3.2–4.5	3-♂
16-♂	>5	6	Adult	Full (proximal humerus fused)	>4.5	22-♂

^1^Based on [49] for crested langurs. Ages are based on gingival emergence times of captured wild crested langurs examined at 3-month intervals, except where noted.

^2^Based on Fairbanks, pers. comm. Ages are from known-age captive individuals in a vervet colony housed at UCLA-VA Vervet Research Center.

^3^Based on crested langurs reaching adult physical strength at about 6 years [49], Francois' langur males reaching reproductive maturity at 5 years [64] as cited in [65] and Phayre's males reaching adulthood at 5 years [66].

**Table 4:** Age class at which skeletal fusions occurs—Males Only. Shading indicates difference between species.

	Langurs	Vervets
Pelvis	Age class at which fusion initiates	
Ischiopubic ramus	2	2
Acetabulum	4	3
Long bones		
Elbow	3	3
Hip	5	4
Ankle	5	3
Knee	5	6
Wrist	5	5
Shoulder	5	5

**Table 5:** Age class at which male adult linear and mass dimensions achieved (*P* < .05). Shading indicates difference between species.

Dimensions	Langurs	Vervets
	Adult dimensions	
Cranial capacity	3	3
Humerus	6	5
Radius	6	5
Femur	5	5
Tibia	6	5
Tail	5	5
Foot	6	5
Trunk height	6	6
Body mass	6	6

**Table:** (a)

	Humerus		Radius		Femur		Tibia	
	Langur	Vervet	Langur	Vervet	Langur	Vervet	Langur	Vervet
Age class 3	98.0 *n* = 7 SD = 10.6	97.5 *n* = 2	92.3 *n* = 7 SD = 9.4	90.5 *n* = 2	124.2 *n* = 7 SD = 13.6	116.5 *n* = 2	109.7 *n* = 7 SD = 11.8	114.0 *n* = 2
Age class 4	114.0 *n* = 3 SD = 8.1	106.0 *n* = 4	109.1 *n* = 3 SD = 7.7	102.8 *n* = 4	145.5 *n* = 3 SD = 10.2	131.8 *n* = 4	128.2 *n* = 3 SD = 8.2	126.0 *n* = 4
Age class 5	132.3 *n* = 2 SD = .8	123.7 *n* = 3 SD = 3.1	124.8 *n* = 2 SD = .7	118.7 *n* = 3 SD = 4.5	170.1 *n* = 2 SD = 5.5	153.7 *n* = 3 SD = 6.8	149.0 *n* = 3 SD = .6	145.3 *n* = 3 SD = 7.2
Age class 6	141.7 *n* = 16 SD = 5.8	127.2 *n* = 17 SD = 4.2	134.3 *n* = 16 SD = 4.6	123.1 *n* = 17 SD = 3.4	177.0 *n* = 16 SD = 7.6	156.1 *n* = 17 SD = 4.6	156.0 *n* = 16 SD = 5.1	147.5 *n* = 18 SD = 4.6

**Table:** (b)

	Foot		Tail		Trunk		Body Mass	
	Langur	Vervet	Langur	Vervet	Langur	Vervet	Langur	Vervet
Age class 3	122.5 *n* = 4 SD = 12.3	115.5 *n* = 2	491.3 *n* = 4 SD = 54.8	435.0 *n* = 2	235.8 *n* = 4 SD = 27.5	249.0	2.0 *n* = 4 SD = .6	2.0
Age class 4	136.5 *n* = 2 SD = 5.0	125.0 *n* = 4	562.5 *n* = 2 SD = 3.5	576.7 *n* = 3	273.5 *n* = 2 SD = 9.2	278.8	2.8 *n* = 2 SD = .2	3.2
Age class 5	154.7 *n* = 3 SD = 5.5	138.0 *n* = 3	681.7 *n* = 3 SD = 15.3	610.0 *n* = 3	337.7 *n* = 3 SD = 15.0	322.0 *n* = 3 SD = 3.5	5.4 *n* = 3 SD = .5	4.6 *n* = 3 SD = .8
Age class 6	161.7 *n* = 12 SD = 4.8	139.9 *n* = 18	707.9 *n* = 12 SD = 30.9	617.0 *n* = 15	366.1 *n* = 12 SD = 11.0	346.7 *n* = 20 SD = 15	6.6 *n* = 12 SD = .7	5.8 *n* = 21 SD = .7

**Table 7:** Traits of male juveniles as a percentage of adult means to highlight the order of body systems maturity^4^.

	Crested langurs (9)			Vervet monkeys (9)		
Body system	Juvenile Mean	Adult Mean	% Growth Completed	% Growth Completed	Adult Mean	Juvenile Mean
Cranial cap. (cc)	64	67	96%	99%	69	68^5^
Foot (mm)	136.3	161.7	84%	91%	139.9	127.2
Tail	570.6	707.9	81%	92%	617	570.7^6^
Femur (mm)	137.1	177.0	77%	87%	156.1	135.7
Humerus (mm)	107.7	141.7	76%	87%	127.2	110
Trunk (mm)	278.1	366.1	76%	83%	346.7	286.6
Body mass (kg)	3.3	6.6	50%	59%	5.8	3.4
Skeleton	27	57	47%	47%	57	27

^4^Only those individuals with data for *all *19 epiphyseal scores, dental scores, long bone lengths and trunk height, and body masses were included, unless otherwise noted.

**^5^** *n* = 8 missing data point for one age class 5 individual

**^6^** *n* = 7; missing data point for one age class 3 and one age class 4 individual.

**Table:** (a) (Each immature age class mean divided by age class 6 (adult) mean)

	Humerus		Femur		Trunk		Body mass	
	Langur	Vervet	Langur	Vervet	Langur	Vervet	Langur	Vervet
Age class 1	nd	37 *n* = 1	nd	36 *n* = 1	36 *n* = 2	35 *n* = 2	7 *n* = 2	7 *n* = 2
Age class 2	46 *n* = 2	nd	44 *n* = 2	50 *n* = 2	42 *n* = 1	52 *n* = 2	7 *n* = 1	17 *n* = 2
Age class 3	69 *n* = 7	77 *n* = 2	70 *n* = 7	75 *n* = 2	64 *n* = 4	72 *n* = 2	30 *n* = 4	35 *n* = 2
Age class 4	80 *n* = 3	83 *n* = 4	82 *n* = 3	84 *n* = 4	75 *n* = 2	80 *n* = 3	42 *n* = 2	55 *n* = 3
Age class 5	93 *n* = 2	97 *n* = 3	96 *n* = 2	98 *n* = 3	92 *n* = 3	93 *n* = 3	82 *n* = 3	79 *n* = 3
Age class 6	100 *n* = 16	100 *n* = 17	100 *n* = 16	100 *n* = 17	100 *n* = 12	100 *n* = 20	100 *n* = 12	100 *n* = 21

**Table:** (b)

	Tail		Foot	
	Langur	Vervet	Langur	Vervet
Age class 1	44 *n* = 2	45 *n* = 2	51 *n* = 2	50 *n* = 2
Age class 2	51 *n* = 1	64 *n* = 2	53 *n* = 1	66 *n* = 2
Age class 3	69 *n* = 4	71 *n* = 2	77 *n* = 4	83 *n* = 2
Age class 4	79 *n* = 2	93 *n* = 3	84 *n* = 2	89 *n* = 4
Age class 5	96 *n* = 3	99 *n* = 3	96 *n* = 3	99 *n* = 3
Age class 6	100 *n* = 12	100 *n* = 15	100 *n* = 12	100 *n* = 18
